# Characteristics of attrition within the SuperMIX cohort of people who inject drugs: a multiple event discrete-time survival analysis

**DOI:** 10.1186/s12874-024-02377-1

**Published:** 2024-10-30

**Authors:** Shady Abdelsalam, Paul A. Agius, Rachel Sacks-Davis, Amanda Roxburgh, Michael Livingston, Lisa Maher, Matthew Hickman, Paul Dietze

**Affiliations:** 1 National Drug Research Institute (NDRI), Faculty of Health Sciences, Curtin University, Melbourne, Australia; 2 Disease Elimination Head, Alcohol and other Drug Research, Burnet Institute, Melbourne, Australia; 3 Faculty of Health, Deakin University, Burwood, Australia; 4 School of Public Health and Preventative Medicine, Monash University, Melbourne, Australia; 5 Specialty of Addiction Medicine, Central Clinical School, Sydney Medical School, The Faculty of Medicine and Health, University of Sydney, Sydney, Australia; 6 National Drug and Alcohol Research Centre, UNSW, Sydney, Australia; 7 Monash Addiction Research Centre, Monash University, Melbourne, Australia; 8 Centre for Alcohol Policy Research, La Trobe University, Bundoora, Australia; 9 The Kirby Institute, Faculty of Medicine, UNSW, Wallace Wurth Building, Kensington, NSW Australia; 10 Population Health Sciences, Bristol Medical School, University of Bristol, Bristol, UK

**Keywords:** People who inject drugs, Cohort studies, Cohort attrition, Lost to follow up

## Abstract

**Background:**

Compared to the general population, people who inject drugs have poor health and wellbeing. Longitudinal studies can provide insight into factors driving these worse health outcomes but are subject to methodological challenges, such as cohort attrition. The aim of this study was to assess and characterise attrition in a prospective cohort of people who inject drugs in Victoria, Australia.

**Methods:**

Using annually collected self-reported data from The Melbourne Injecting Drug User Cohort Study (SuperMIX) from September 2008 to January 2021, we estimated the incidence of participants being lost-to-follow-up (LTFU), with an episode of being LTFU defined as participants not undertaking a follow-up interview within two years of their last interview. We utilised a multiple event discrete-time survival analysis on participant period-observation data to estimate the associations between key factors and LTFU. Key areas of exposure measurement in analyses were sociodemographic, drug use and mental health.

**Results:**

A total of *n* = 1328 SuperMIX participants completed a baseline interview, with *n* = 489 (36.8%) LTFU, i.e. not completing a follow-up interview in the following two years. Increased attrition was observed among SuperMIX participants who were: born outside Australia, younger than 30 years, reporting having completed fewer years of education, not residing in stable accommodation, not in stable employment and not on opioid agonist therapy (OAT).

**Conclusions:**

The attrition rate of the SuperMIX cohort has largely been stable throughout the duration of the study. Higher attrition rates among individuals at greater sociodemographic disadvantage and not on OAT suggest that additional efforts are required to retain these participants. Findings also suggest that SuperMIX might not be capturing data on adverse health and wellbeing outcomes among subpopulations at high risk of harm.

**Supplementary Information:**

The online version contains supplementary material available at 10.1186/s12874-024-02377-1.

## Background

People who inject drugs often experience a higher prevalence of chronic disease, premature mortality and poorer overall health and wellbeing, compared to the general population [1]. Longitudinal studies provide a platform for understanding cause-and-effect relationships between these factors not available from cross-sectional studies. Previous prospective observational studies of people who inject drugs have been used to highlight the protective effects of interventions such as opioid agonist therapy (OAT) and needle and syringe programs on outcomes such as hepatitis C virus acquisition [2, 3]. However, a major challenge with longitudinal studies is cohort attrition, where participants are at risk of being lost-to-follow-up (LTFU) after enrolment and to each follow-up wave.

Cohort participants being LTFU can result in a form of selection bias, which can reduce the generalisability and validity of study findings [4, 5]. Population studies have suggested that non-death attrition, such as participants being LTFU, results in more significant bias than participant death [6]. Factors associated with attrition in general population studies include increasing age and decreased cognitive function [7]. More specifically, factors associated with attrition in previous cohort studies of people who inject drugs include recruitment location, incomplete contact information, male gender, previous diagnosis of depression, recent incarceration and unstable housing [8–10]. Strategies to address attrition in cohort studies include: downstream post hoc methodological approaches such as inverse probability weighting for marginal structural models [11]; and upstream a priori research design and recruitment methods [12] such as comprehensive follow up protocols, utilising advertisements, ongoing phone contact, social media and GPS tracking [5, 12–14]. These latter strategies can be costly and are not always reliable when following up highly mobile cohorts such as people who inject drugs.

Understanding the determinants of attrition in cohorts of people who inject drugs may improve understanding and generalisability of study findings, inform analytic approaches to reduce potential biases, and help to develop strategies to prevent LTFU among cohorts in the first instance. Stewart et al. [13] assessed factors impacting attrition in an Australian cohort of men with a history of injecting drug use, who had been released from prison. Individuals were interviewed at baseline, 3 months, 6 months and 12 months after their release, with individuals completing one post-release interview described as “retained” and those missing all post-release interviews “LTFU” [13]. Overall, 85% of participants completed at least one of three follow up interviews over a two year study period; younger age was the only baseline characteristic that distinguished retained participants from those LTFU [13]. In a longitudinal cohort study of heroin dependent individuals (many of whom injected drugs) recruited from treatment services, Teeson et al. employed a range of follow-up techniques and reported a retention rate of 76.3% of individuals at two years and 70.1% of participants completing a follow-up interview at 11 years [9]. Male participants and individuals with a diagnosis of major depression at baseline were reported to be independent predictors of being LTFU after 11 years [9]. How these findings relate to broader populations of people who inject drugs, who may not be in treatment, is unknown.

The Melbourne injecting drug user cohort study (SuperMIX) is a prospective observational study of people who inject drugs in Melbourne, Australia. Established in 2008, SuperMIX is Australia’s largest and only active prospective cohort study of people who inject drugs [15]. Data from the SuperMIX cohort has helped inform the literature regarding a broad range of outcomes, including non-fatal overdose [16], ambulance attendance [17], risky injecting practices [18], psychological distress [19] and oral health related quality of life [20]. SuperMIX data have also informed and contributed to the evaluation of harm reduction interventions, such as the Medically Supervised Injection Room (MSIR), Melbourne’s first supervised injecting facility [21–23].

The initial SuperMIX cohort profile in 2013 (*n* = 688), with data collected from 2008 to 2010, reported a 71% participant retention rate at one year [8]. Correlates of attrition after 12 months included recruitment location within Central and Inner West Melbourne, male gender and incomplete contact information [8]. The second SuperMIX cohort profile (*n* = 1303), reviewing data from 2008 to 2019 and published in 2022, revealed a 2-year retention rate of 68%, with 32% of participants not having a follow-up interview in the two years preceding 1 July 2019. Being LTFU was associated with younger age, not identifying as Aboriginal or Torres Strait Islander, being born outside Australia and residing in more stable housing [15].

Despite a limited understanding of the incidence and correlates of attrition in the SuperMIX cohort, attrition rates beyond the first two years of follow-up has not been studied. In addition, there has been no analysis of multiple LTFU events among this cohort. Subsequently, a gap exists with regards to the factors associated with both long-term and multiple LTFU events within the cohort. This study is an important precursor to a broader program of work utilising the SuperMIX cohort to investigate the impact of MSIR use on health outcomes for people who inject drugs. Understanding the correlates of attrition in the cohort will provide important foundations for these future studies.

In this study we aimed to:

1) Assess the SuperMIX cohort attrition rate, over the period from 2008 to 2021, observing multiple event LTFU for participants.

2) Characterise attrition within the SuperMIX cohort for multiple event LTFU.

## Methods

### Study sample

This study uses primary data from interviews conducted with participants from the SuperMIX cohort, established in 2008 to assess trajectories of injecting drug use in Melbourne, Victoria [8]. The eligibility criteria and recruitment processes relevant to SuperMIX are discussed in detail elsewhere [8, 15]. Briefly, sampling methods for recruitment included respondent-driven sampling, snowball sampling and street outreach, where interviewers attended recruitment locations and eligible participants were recruited through flyers and word of mouth [8]. Inclusion criteria were being over 18 years of age, monthly injecting drug use in the six months prior to baseline interview, living in the Greater Melbourne or Geelong region, having a valid Medicare (Australia’s universal healthcare system) card and providing detailed contact information, entailing full name, address and telephone number, any aliases and contact details for significant others who may be able to provide new contact details if these change for participants. Email addresses and social media details have also been collected since 2015 [8, 15].

SuperMIX interviews involve a quantitative annual questionnaire, conducted in person or on the phone [15]. All participants provided informed consent prior to their interview. Up to January 2021, *n* = 1328 participants had completed a baseline interview. The SuperMIX study has been funded by the Colonial Foundation Trust and the Australian National Health and Medical Research Council (NHRMC) (Grant Numbers: 545891 & 1126090). Ethical approval for the study was obtained from Human Research Ethics Committees at the Victorian Department of Health and Human Services, Victorian Department of Health and the Alfred Hospital (Ethics approval number 599/21).

### Follow-up

SuperMIX participants are followed-up annually, with a multitude of strategies utilised to prevent cohort attrition. These strategies include frequent contact prior to the follow-up appointment, contacting participants’ known friends or relatives, flexibility around follow-up appointment dates, providing physical follow-up cards, correspondence via mail and electronic mechanisms, such as email and Facebook handles [8, 15].

For the purposes of the analyses in this study, participants were defined as being LTFU once they had missed a follow-up interview for two or more years (from either their baseline or last follow-up interview) until either (1) the participant returned to complete another cohort interview (and became at risk of another LTFU period); or (2) did not return to complete any subsequent interviews and were censored as LTFU. Given the nature of the study design (annual interviewing), time was treated as discrete (i.e. yearly person-period observations). For individuals with multiple LTFU events, time was restarted from the date of an individual’s return SuperMIX interview.

### Outcome

The outcome of interest was participants being LTFU. Given the prospective nature of the SuperMIX cohort and length of follow up in this study, with data available from 2008 to 2021, participants could have (or were at risk of having) multiple LFTU events, where they completed a follow-up interview after the 2-year definitional period (i.e. they return to the study after a period of LTFU to complete another study interview). We chose not to censor participants who returned to the study after their first episode of LTFU, to appropriately model this participant movement in and out of study contact. Participants were censored either at the study censor date or if they were LTFU and did not return to the study. The censor date and two-year follow up period used in this study to define LTFU mean that participants recruited after January 10, 2021, were not included.

### Covariates

Both time-invariant and time-varying covariates included in this study were selected a priori based on findings from earlier papers assessing cohort retention rates for people who inject drugs [8, 9, 13]. For the purposes of this study, cohort period (2008–2010, 2011–2013, 2014–2016, 2017–2019, 2020–2021) was a time varying covariate, based on participants’ interview date, allowing for observations of individuals who had been LTFU and returned to the cohort to be included in the analyses. Time-invariant covariates included at baseline were sex (Male versus Female), birthplace (Australia versus Outside Australia), Aboriginal and Torres Strait Islander (Yes versus No), age range (18–30, 30–39 and 40 + years). Time-varying covariates included accommodation status (stable vs. unstable), as defined by Whittaker et al. [24]. Stable accommodation entailed participants living in a privately-owned, rental or parents’ residence; conversely, unstable accommodation involved participants who were homeless, in boarding houses, hostels, shelters and/or couch surfing [24]. Geographic interview sites within Victoria (CBD, St Kilda and Inner suburbs, Western suburbs and Geelong, Eastern Suburbs and Mornington Peninsula and Phone or Outreach) was another time-varying covariate assessed. Phone or outreach interviews were conducted as follow up interviews for participants who had already completed a face-to-face baseline interview. Other time-varying covariates included education (Lower than Year 10, Year 10–11 and Year 12, higher or other secondary qualification), and employment (Yes versus No).

Drug use and other risk factor covariates included total drug injections in the past week (None, 1–7 and 8 or more), current OAT (Yes versus No) and history of ever being arrested (Yes versus No). Mental health was assessed via the short form 8 quality of life scale (SF8) mental health component summary (MCS) score [8, 25]. The SF8 and MCS are described in further detail elsewhere [25]. Similar to an earlier study assessing MCS scores amongst SuperMIX participants [26], MCS scores were categorised into (low, average and high), corresponding to the lower quartile, average (25–75%) and upper quartile of SuperMIX sample scores. Lower MCS scores are indicative of worse mental health status.

### Statistical analyses

Multiple event discrete-time survival analyses, using generalised linear mixed modelling (GLMM, complementary log-log link function, binomial distribution), were undertaken on person-period data to estimate associations between participant LTFU and key sociodemographic, drug use and mental health factors. Given the dependency in the outcome (i.e., multiple event LTFU, level-1), the GLMM’s specified a random intercept for study participant (level-2). The GLMMs approximate a Cox regression analyses in discrete time and produce hazard ratios (HR) and 95% Confidence Intervals (CIs) for the associations between multiple event LTFU and the covariates of interest. Statistical models were adjusted for both time invariant and time-varying covariates. Estimates from discrete-time models were offset for the log time between interviews. Post-estimation joint Wald tests were used to provide inference for polytomous factors in the modelling, including interaction terms estimated to model the time-dependence in the hazards of key covariates. Associations were considered statistically significant at 5%. A complete case approach was used to account for missing data in our the GLMMs. All analyses were performed using the Stata 17/SE statistical software package (Statacorp LLC, Texas, USA).

## Results

### Study population

Summary characteristics of the SuperMIX cohort at baseline (*n* = 1328) are presented in Table 1. The mean age was 32.4 years (SD = 9, median 30 years), with 68% of participants male and 32% female. Most participants were born in Australia (*n* = 1097, 83%) and 12% identified as Aboriginal or Torres Strait Islander. Over a third of participants reported residing in unstable accommodation. Baseline interview locations were relatively dispersed across sites in metropolitan Melbourne and Geelong. Approximately 40% of participants had completed Year 12 or higher at school and the significant majority (87%) of respondents reported being unemployed. Almost half the cohort (48%) reported injecting 1–7 times in the previous week and 40% reported eight or more episodes of injecting drug use in the previous week. Over a third of participants (36%) reported being on OAT. Almost half (48%) the cohort had SF-8 mental health component summary scores within the “average” (25–75%) range, 24% in the “high” (> 75%) range and 28% in the “low” (< 25%) range.

**Table 1:** Selected baseline summary characteristics (*n* = 1328)

	*n* (%)
**Sex**	
Female	430 (32)
Male	893 (68)
**Age**	
Mean age (SD)	32.4 (9)
< 30	622 (47)
30–39	445 (33)
> 40	261 (20)
**Ethnicity**	
Born in Australia	1097 (83)
Born overseas	228 (17)
**Aboriginal and Torres Strait Islander**	
No	1162 (88)
Yes	163 (12)
**Accommodation status**	
Stable	846 (64)
Unstable	473 (36)
**Interview site**	
CBD, St Kilda and Inner suburbs	546 (41)
Western suburbs and Geelong	510 (38)
Eastern Suburbs and Mornington Peninsula	272 (21)
**Highest level of education**	
< Year 10	364 (27)
Year 10–12	408 (31)
Year 12/Higher/Other	555 (42)
**Employment**	
No	1148 (87)
Yes	177 (13)
**Frequency of injecting drug use in the past week**	
None	152 (11)
1–7	633 (48)
≥ 8	535 (41)
**Current opioid agonist therapy (OAT)**	
No	843 (63)
Yes	485 (37)
**History of arrest**	
No	390 (30)
Yes	915 (70)
**SF8 MCS score**	
< 31 “Low” (Lower quartile < 25%)	357 (28)
31–52 “Average” (25–75%)	620 (48)
>=53 “High” (Upper quartile > 75%)	313 (24)

### Incidence of attrition

**Fig. 1:**
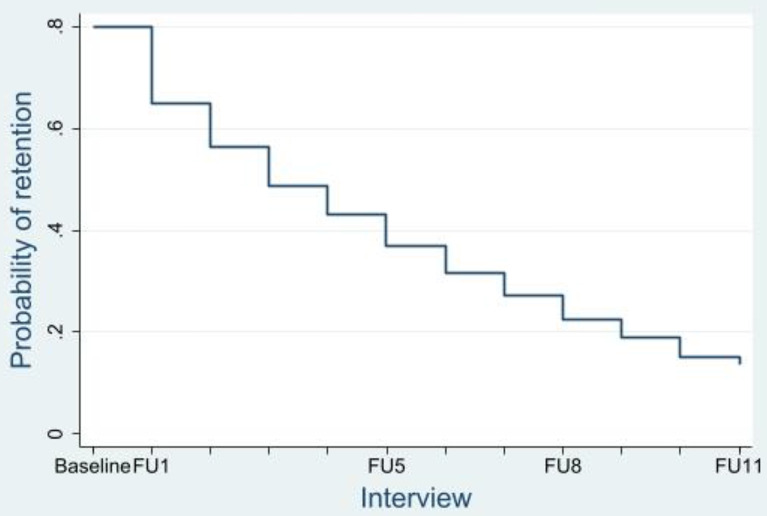
Predicted probability of retention in the SuperMIX study. FU: Follow up

The total number of LTFU events was *n* = 1461, out of *n* = 5476 observations for all participants. These *n* = 1461 LTFU events entailed 1121 (77%) first time LTFU events and 340 (23%) repeat LTFU events. Incidence rates for multiple event LTFU, which were calculated by estimating the predicted means for the discrete time periods used in the analyses, are reported in Table 2. The predicted survival for participants is plotted in Fig. 1. Within SuperMIX, *n* = 489 (36.8%) of participants had a single episode of being LTFU after their baseline interview, with *n* = 839 (63.2%) completing a follow up interview within two years of their baseline interview. Highest incidence of attrition was observed at baseline, with an incidence rate of 38.4 per 100 person years (PY) (95%CI: 36.2,40.6). This steadily decreased to the lowest incidence of attrition of 17.4 per 100PY (95%CI: 13.4,21.5), observed after participants’ fourth follow up interview. Incidence rates of attrition subsequently increased, reaching 25.1 per 100PY (95%CI: 19.1,31) after participants’ seventh follow up interview.

**Table 2:** Incidence rate of attrition in the SuperMIX cohort

Time period	% of participants LTFU	Incidence rate (per 100 PY)	95% CI
Baseline	36.8	38.4	36.2,40.6
Follow up 1	28.8	31.4	28.5,34.2
Follow up 2	19.7	21.4	18.2,24.7
Follow up 3	19.2	18.6	14.9,22.2
Follow up 4	18.2	17.4	13.4,21.5
Follow up 5	20.6	20.7	16,25.3
Follow up 6	24.2	22.9	17.6,28.2
Follow up 7	28.3	25.1	19.1,31
Follow up 8	30.8	23.1	16.5,29.8
Follow up 9	24.2	23.2	15.4,31
Follow up 10	21.3	22.2	13,31.4
Follow up 11	22.5	21.6	8.8,34.3

### Factors associated with study attrition

The results from discrete-time survival analyses are shown in Table 3. The cohort period for participants was associated with attrition (χ2 [4] = 83.03, *P* < 0.001). Compared to SuperMIX participants interviews from 2008 to 2011, participants interviewed between 2014 and 2016 (aHR 1.58, 95%CI: 1.26,1.98; *P* < 0.001), 2017–2019 (aHR 2.18, 95%CI: 1.8,2.64; *P* < 0.001) and 2020–2021 (aHR 2.06, 95%CI 1.56,2.72; *P* < 0.001) had 1.6, 2.2 and 2.1 times the risk of multiple event LTFU, respectively. SuperMIX respondents born outside Australia were more likely to be LTFU (aHR 1.19, 95%CI: 1.03,1.37; *P* = 0.02), compared to Australian-born respondents. Age was independently associated with attrition (χ2 [2] = 7.86, *P* = 0.02). Participants aged 30–39 (aHR 0.84, 95%CI: 0.73,0.97; *P* = 0.02) and 40 and above years (aHR 0.77, 95%CI: 0.63,0.94; *P* = 0.01) exhibited a lower risk of being LTFU, compared to participants lower than 30 years of age.

**Table 3:** Multiple-event generalised linear mixed model* (multi-level discrete-time survival analysis) showing associations between LTFU and sociodemographic, drug use and mental health factors: adjusted hazard ratio (aHR), 95% confidence interval (95% CI) and probability value (p-value) *n* = 1328

Variables	aHR	95%CI	*P*-value
**Cohort period**			
2008–2010	**Ref.**		
2011–2013	1.04	0.83,1.3	0.72
2014–2016	1.58	1.26,1.98	< 0.001
2017–2019	2.18	1.8,2.64	< 0.001
2020–2021	2.06	1.56,2.72	< 0.001
**Sex**			
Female	**Ref.**		
Male	1.05	0.93,1.18	0.4
**Birthplace**			
Australia	**Ref.**		
Overseas	1.19	1.03,1.37	0.02
**Aboriginal and Torres Strait Islander**			
No	**Ref.**		
Yes	0.93	0.77,1.12	0.45
**Age at baseline interview**			
< 30	**Ref.**		
30–39 years	0.84	0.73,0.97	0.02
40 + years	0.77	0.63,0.94	0.01
**Accommodation status**			
Stable	**Ref.**		
Unstable	1.15	1.02,1.28	0.02
**Interview site**			
CBD, St Kilda and inner suburbs	**Ref.**		
Western suburbs and Geelong	1.09	0.95,1.25	0.2
Eastern suburbs and Mornington Peninsula	0.98	0.84,1.16	0.84
Phone/outreach	1.45	1.11,1.9	0.01
**Education**			
< Year 10	**Ref.**		
Year 10–11	0.85	0.74,0.99	0.03
Year 12/Higher/Other	0.67	0.59,0.77	< 0.001
**Employment**			
Yes	**Ref.**		
No	1.18	1.01,1.38	0.03
**Total drug injections in the past week**			
None	**Ref.**		
1–7	0.97	0.84,1.12	0.67
8+	0.93	0.8,1.09	0.39
**Current OAT**			
Yes	**Ref.**		
No	1.17	1.05,1.32	0.01
**History of arrest**			
No	**Ref.**		
Yes	0.98	0.87,1.1	0.75
**MCS summary score**			
< 31(Lower quartile < 25%)	**Ref.**		
31–52(25–75%)	1.05	0.92,1.2	0.44
>=53 (Upper quartile > 75%)	1.15	0.99,1.34	0.07

OAT: Opioid agonist therapy. MCS: SF8 mental health component summary

*The statistical model also included terms for the baseline hazard and was offset for the duration of time between follow-up interviews. The model utilised multiple event discrete-time survival analyses, using generalised linear mixed modelling (GLMM, complementary log-log link function, binomial distribution), to estimate associations between participant LTFU and key sociodemographic, drug use and mental health factors

Time-invariant covariates included in this model were sex, birthplace, Aboriginal and Torres Strait Islander, age range and history of arrest. Time-varying covariates included cohort period, accommodation status, interview site, education, employment, total drug injections in the past week, current OAT and mental health component summary (MCS) score

**Fig. 2:**
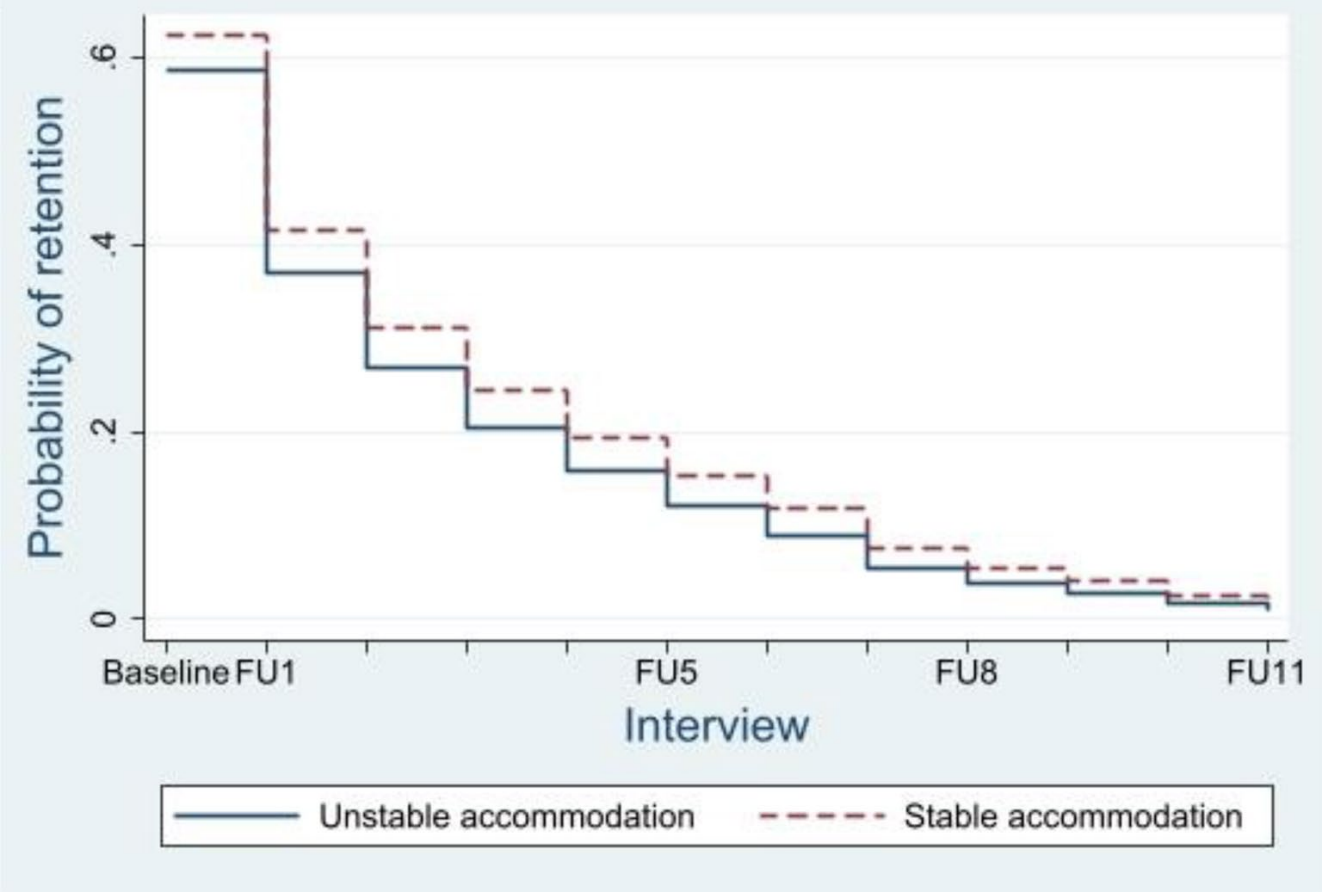
Predicted probability of SuperMIX retention for participants in stable versus unstable accommodation. FU: Follow up

**Fig. 3:**
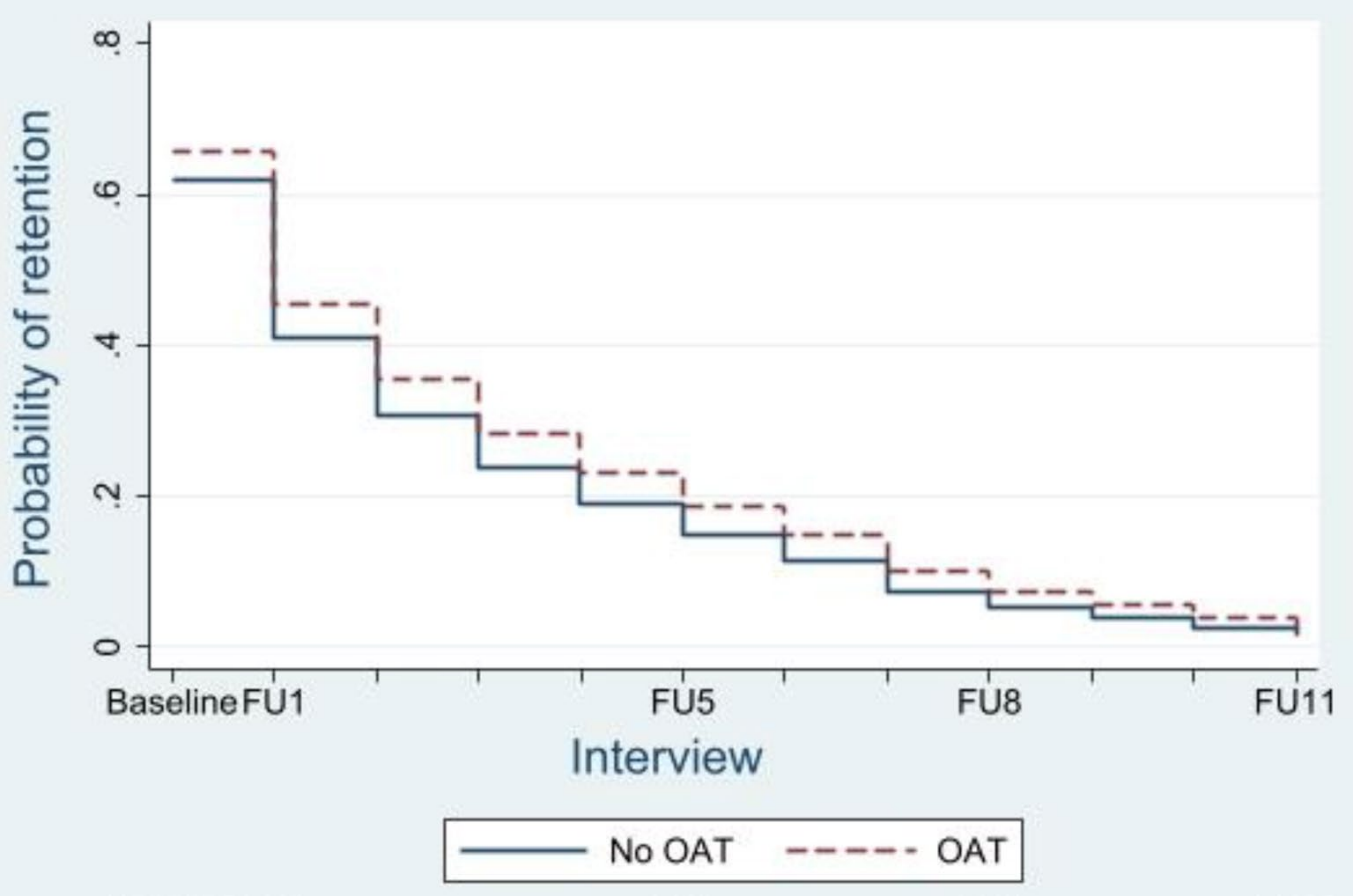
Predicted probability of SuperMIX retention for participants on OAT versus not on OAT. FU: Follow up

Cohort members who were in unstable accommodation were at increased risk of being LTFU (aHR 1.15, 95%CI: 1.02,1.28; *P* = 0.02), compared to those in stable accommodation. This is highlighted by Fig. 2, which shows the probability of study retention between participants in stable versus unstable accommodation. Participants’ years of education attained were correlated with attrition (χ2 [2] = 33.63, *P* < 0.001). Individuals who had completed Year 10–11 (aHR 0.85, 95%CI: 0.74,0.99; *P* = 0.03) or higher (aHR 0.67, 95%CI: 0.59,0.77; *p* < 0.001) exhibited a lower risk of multiple event LTFU, compared to individuals who had completed fewer years of education. Interview site was correlated to attrition from the study (χ2 [3] = 9.58, *P* = 0.02). Participants conducting SuperMIX interviews via phone or outreach services were at increased risk of attrition (aHR 1.45, 95%CI: 1.11,1.9; *P* = 0.01), compared to face-to-face interviews in Melbourne’s CBD, inner suburbs and St Kilda. Respondents who were unemployed were at higher risk of attrition (aHR 1.18, 95%CI: 1.01,1.38; *P* = 0.03), compared to those in stable employment. SuperMIX participants not on OAT (aHR 1.17, 95%CI: 1.05,1.32; *P* = 0.01) were at increased risk of being LTFU, compared to participants on OAT at the time of interview. Figure 3 illustrates the predicted survival of SuperMIX participants on OAT, versus participants not on OAT. Interaction terms were estimated to model the time-dependence in the hazards of key covariates. Details of these interaction terms are provided in the attached supplementary material.

## Discussion

Within the SuperMIX cohort of people who inject drugs in Melbourne, Australia we detected a baseline attrition rate of 36.8%, with 63.2% of participants completing a follow up interview within the two years prior to the censor date (January 10, 2021). This is higher than the 23.7% attrition rate reported for a cohort of heroin dependent people in Australia [27], but compares favourably with 47.8% and 55% two-year attrition rates reported by cohorts of people who inject drugs in Sweden [28] and Malaysia [29], respectively. One correlate of multiple event LTFU was participants’ last interview being later into the SuperMIX study, specifically in the period from 2014 onwards. Other correlates of attrition were consistent with previous research with younger age [15, 28, 30] and being born overseas [15] associated with increased risk of attrition. Time-varying correlates of attrition in our study also largely mirrored other studies of people who inject drugs, with fewer years of education [28, 29, 31], residing in unstable accommodation [32], being unemployed [31] and not being on OAT [28] all associated with LTFU.

Our attrition incidence findings are largely consistent with the most recently published SuperMIX cohort profile in 2022, which had a censor date of 1 July 2019 [15], and the original cohort profile published in 2013 [8]. Further, the attrition correlates were similar to the 2022 study which reported a positive association between younger age and being born outside Australia and being LTFU [15]. However, the 2022 cohort profile also reported that participants LTFU were less likely to identify as Aboriginal or Torres Strait Islander or to reside in unstable accommodation [15]. This contrasts with findings from the current study, where SuperMIX respondents residing in unstable accommodation were at increased risk of attrition, compared to respondents in stable accommodation. The finding in the 2022 cohort profile appears anomalous, potentially driven by a biased simple cross-sectional analytic approach, with our current longitudinal analysis, with a multiple event outcome, more in line with expected effects of accommodation status on attrition.

Our findings also contrast with correlates of attrition reported in the 2013 SuperMIX cohort profile, which found recruitment from Inner West and Central Melbourne (inner-urban sites with previously identified illicit drug markets), male gender and providing inadequate contact details with being LTFU at the 12-month follow up interview [8]. We found that participants who completed phone or outreach interviews had increased risk of attrition, compared to participants attending in-person interviews. These findings are not surprising, as participants interviewed by phone have typically moved away from Melbourne and the drug scene in which they were recruited. This makes follow-up through, for example, word of mouth contact, more difficult. Similarly, individuals interviewed in outreach locations are more likely to be in unstable accommodation, which was associated with LTFU.

Our analyses illustrate that the correlates of attrition within the SuperMIX cohort are primarily sociodemographic in nature, with no significant effects of patterns of drug use, other than not being on OAT. People who inject drugs are at increased risk of harms from their exposure to adverse risk environments, with subsequent higher risks of arrest, incarceration, homelessness and bloodborne virus acquisition and transmission [33]. Our study found a higher risk of attrition among those experiencing more significant marginalisation, such as participants with lower educational achievement and residing in unstable accommodation. One potential implication of our overall findings around marginalisation is that as time goes by and SuperMIX cohort participants potentially face more difficult living circumstances, they are LTFU, with the subsequent social inequities and burden of disease they experience not being adequately captured. Likewise, while we found that being on OAT was protective against attrition, similar to other cohort studies of people who inject drugs [28], the implication of this finding is that the SuperMIX study may not fully capture the experiences of those not engaged in OAT. This study aims to inform future research into the impact of MSIR on health outcomes in people who inject drugs. With MSIR frequently utilised by individuals at greater socioeconomic disadvantage [22], it can potentially serve as a location for targeted outreach efforts to improve cohort retention amongst subpopulations experiencing marginalisation.

Our study makes an important contribution to the literature with respect to understanding attrition in longitudinal research of outcomes for people who inject drugs. Specifically, the results from our study, where we identify factors which either increase the hazard or are protective of a study participant being lost-to-follow-up, are important from a methodological perspective. Here, the application of more advanced statistical modelling estimation techniques (e.g. maximum likelihood) on longitudinal data exposed to attrition bias often require the inclusion of auxiliary variables (i.e. associated with missingness but not important to theoretical model of interest) in statistical modelling to increase the likelihood of assumption plausibility, for the less restrictive (ignorable) ‘missing-at-random’ (MAR) missing data process.

The findings from our study provide some direction for which variables might be important in this process and may help guide the composition of statistical models and stochastic missing data imputation to provide unbiased estimation in research pertaining to people who inject drugs. Further, other cohort studies with similar populations might also consider adopting the statistical modelling approach outlined in this paper to estimate multiple-event LTFU and the factors associated with attrition – identifying study-specific factors associated with attrition in their data, which might in turn be important in the application of appropriate missing data treatments, ensuring ignorability assumptions have maximised likelihood.

### Limitations

Data for this study were self-reported, which potentially carries a risk of recall and other bias. However, self-reported data from people who inject drugs has been found to provide a valid method for description of drug use and drug-related problems [34]. Our measure of wellbeing, the SF8, is limited in its scope with regards to reporting mental health conditions and is generally used as a measure of individuals’ general wellbeing [25], rather than a screening tool for mental illness.

## Conclusion

Within the SuperMIX cohort of people who inject drugs, we examined the cohort attrition rate and multiple LTFU events over a 13-year period, from 2008 to 2021. We detected an attrition rate of 36.8%, with 63.2% of SuperMIX respondents completing a follow up interview within the two years of their baseline interview. In characterising attrition in the cohort, we found that sociodemographic marginalisation was related to attrition, consistent with findings from other cohort studies. This suggests that sociodemographic factors need to be included in attempts to adjust for cohort attrition when examining outcomes for people who inject drugs using prospective observational data. Further studies are needed to better understand any potential causal effects of the associations reported in this study.

## Electronic supplementary material

Below is the link to the electronic supplementary material.

Supplementary Material 1

## Data Availability

The data underlying this article cannot be shared publicly, due to ethical and privacy limitations. Researchers interested in data access are advised to contact the corresponding author, Professor Paul Dietze, to request the application form for data access developed for the SuperMIX project.
