# Supplementary material for: Characteristics of attrition within the SuperMIX cohort of people who inject drugs: a multiple event discrete-time survival analysis
Source: BMC Med Res Methodol. 2024 Oct 30;24:257. doi: 10.1186/s12874-024-02377-1 (PMC11523591; doi:10.1186/s12874-024-02377-1)
Supplement: Supplementary file 1 — Supplementary Material 1 [file 12874_2024_2377_MOESM1_ESM.docx]

**Supplementary material**

Random intercept models were fitted, with interaction terms estimated to model the time-dependence in the hazards of key covariates (i.e. proportional hazards assumption). These interaction terms were not statistically significant. Post-estimation Wald tests for these interaction terms were performed and the results are reported in the table below.

**Supplementary table S 1. Post-estimation Wald tests to model the time-dependence in the hazards of covariates**

| **Variables** | **Wald test statistic** | **P-value** |
| --- | --- | --- |
| Sex | χ2 (10) = 8.47 | 0.58 |
| Birthplace | χ2 ( 11) = 5.43 | 0.91 |
| Aboriginal and Torres Strait Islander | χ2 ( 11) = 9.78 | 0.55 |
| Age at baseline | χ2 ( 20) = 26.42 | 0.15 |
| Accommodation | χ2 ( 11) = 11.15 | 0.43 |
| Interview site | χ2 ( 32) = 35.93 | 0.29 |
| Highest level of education | χ2 ( 20) = 8.86 | 0.98 |
| Employment | χ2 ( 11) = 4.61 | 0.95 |
| Frequency of injecting drug use in the past week | χ2 ( 22) = 27.83 | 0.18 |
| Current opioid agonist therapy (OAT) | χ2 ( 11) = 3.66 | 0.98 |
| History of arrest | χ2 ( 10) = 9.03 | 0.53 |
| SF8 MCS score | χ2 ( 22) = 19.69 | 0.6 |
